# Elevated oleic acid serum concentrations in patients suffering from alcohol dependence

**DOI:** 10.1186/2049-9256-1-13

**Published:** 2013-08-23

**Authors:** Annekatrin Teubert, Johannes Thome, Andreas Büttner, Jörg Richter, Gisela Irmisch

**Affiliations:** Clinic and Policlinic for Psychiatry and Psychotherapy, University of Rostock, Gehlsheimerstr. 20, 18147 Rostock, Germany; Department of Forensic Medicine, University of Rostock, St.-Georg-Str. 108, 18055 Rostock, Germany; College of Medicine, Swansea University, Singleton Park, Swansea, SA2 8PP UK; Norway Centre for Child and Adolescent Mental Health, Eastern and Southern Norway, P.O. Box 4623, Oslo, 0405 Norway

## Abstract

**Background:**

Alcohol-induced damages such as brain atrophy and fatty liver are closely related to a disturbed lipid metabolism. In animal models, a linkage between chronic alcohol consumption and changes in fatty acid (FA) composition in various organs and cells is well known and there is some indication that this phenomenon could be linked to behavioural alterations associated with alcohol addiction such as craving. However, the influence of ethanol on secretory FA has not been investigated so far. In this study, we therefore aimed at investigating whether there is a significant change of serum FA composition in patients suffering from alcohol dependence. We compared patients before and after treatment (detoxication) with control individuals who did not suffer from addiction. The roles of age, the duration and intensity of alcohol use and lifestyles were considered.

**Methods:**

Serum FA was measured in 73 male ethanol dependent patients before and after alcohol withdrawal in an in-patient setting. Additionally, of this group, 45 patients were matched with 45 healthy male volunteers as controls.

**Results:**

We found significant differences in the FA composition before and after detoxication as well as between patients and controls. After detoxication, the values changed towards the ones in healthy controls. The main finding during acute alcohol use was an increased oleic acid concentration above the level of the linoleic acid concentration.

**Conclusions:**

An elevated oleic/linoleic acid ratio seems to be a state marker for acute alcohol use and may be a relevant trait marker during detoxification and possibly the subsequent therapeutic measures. The results of this pilot study need to be replicated in a larger study also including female patients. Further, the specificity of this potential biomarker needs to be determined.

## Background

Recently, the effect of the dose-dependent balance between antioxidative properties of polyphenoles contained in many alcoholic beverages and the pro-oxidative effects of alcohol itself has been discussed controversially [1, 2]. Pro-oxidative vascular injuries are seen after heightened alcohol consumption [3]. Pathological alcohol consumption also often leads to hepatic diseases (fatty liver, liver cirrhosis) which in turn may cause a disturbed metabolism of fatty acids (FA) and phospholipids [4].

The influence of chronic alcohol use on the FA metabolism is not yet fully understood. Changes of the FA composition in various organs were found in studies based on animal models after chronic alcohol abuse [5–8]. Human studies are rare, and their findings, often based on relatively small sample sizes, remain inconclusive [9]. Possible benefits of the supplementation with polyunsaturated FAs were investigated in a group of abstinent patients [10, 11] and an increased microviscosity and fluidity were found in erythrocyte membranes of patients after chronic alcohol use [12]. The peroxidation of lipids in patients could indicate an injury of membranes by oxidative stress [13]. An alcohol-induced overproduction of nitric oxide, which reacts with superoxide radicals to synthesise peroxynitrite, might also represent one of the many possible causes for the membrane damage [14].

The cannabinoid system may represent another link between alcoholism and FAs which are precursors of endogenous cannabinoids (FA amides). These may play a crucial role in the development of drug or alcohol induced addictive behaviour [15]. In this context, it has been shown recently that the consumption of even a moderate amount of red wine reduces the concentrations of plasma endocannabinoids, anandamide and 2-arachidonoylglycerol [16].

Chronic alcohol use also leads to the downregulation of the cannabinoid 1 receptor function. The synthesis of the endogenous cannabinoid 1 receptor agonists arachidonylethanolamide and 2-arachidonylglycerol is increased by abstinence after chronic alcohol use, and it blocks the deletion of the cannabonoid 1 receptor resulting from excessive alcohol drinking [17, 18]. A previous post-mortem study of the brains of patients suffering from alcoholism revealed that the activity of the endogenous cannabinoid system may be influenced by the type of alcoholism according to the Cloninger classification: Anandamide, dihomo-gamma-linolenoyl ethanolamide and docosahexaenoyl ethanolamide levels were significantly lower when compared to controls especially in Cloninger’s type 1 alcoholics [19, 20].

Further, there are pilot studies looking into the possibility of using FA ethylic esters in the hair of individuals in order to detect possible alcohol dependence [21, 22].

However, the influence of ethanol on secretory FA has not been investigated so far. Thus, the aim of this study was to investigate whether there is a significant change of serum FA composition in patients suffering from alcohol dependence.

## Methods

### Patient and control samples

Study I: 45 alcohol-dependent patients were included in this naturalistic study. They voluntarily attended an inpatient detoxification program at the Department of Psychiatry, University of Rostock. Diagnoses were made by an experienced psychiatrist according to ICD-10 criteria in a clinical routine situation (without the use of standardised psychometric instruments such as structured interviews). The patients were matched for age (± 1 year) and gender (all were male) with healthy controls (for the demographic data of the samples see Table 1).

**Table 1:** **Demographic data of the patient and control groups**

	Study I						Study II
	Alcoholic patients	Controls	Wilk’s λ	P	η.^2^	Power	Alcoholic patients
	N = 45	N = 45					N = 73
Age (x ± sd)	40.56 ± 13.82	40.67 ± 13.80	0.95	0.133	0.051	0.322	46.41 ± 6.07
BMI (x ± sd)	23.93 ± 3.42	25.46 ± 4.01	0.92	0.092	0.083	0.391	24.62 ± 3.88
Alcohol units per day (x ± sd)	17.27 ± 8.90	1.00 +-	0.23	<0.001	0.775	1.00	13.90 ± 9.02
		0.00					
Years of alcohol abuse (x ± sd)	8.97 ± 6.84						9.45 ± 6.41
Concentration of breath alcohol (‰)	0.94 ± 0.98						1.29 ± 1.14
N without any breath alcohol (%)	32.40						30.10
Days of in-patient treatment (x ± sd)	10.82 ± 3.01						10.68 ± 2.58
		Related samples Wilcoxon Rank test					
Smoker %	94.1	22.2	P = 0.002				90.4
Vegetarian %	0.0	2.2	(n for controls is only 1)				0.0
Fish diet %							
Never	14.7	16.7					12.5
≤ 2 times a week	67.6	66.7					80.6
< 2 times a week	17.6	16.7	P = 1.00				6.9

Study II: 73 alcohol-dependent patients within the same setting as described above were investigated at admission and on the day of discharge.

The study protocol was approved by the local ethics committee and was conducted in accordance with the Declaration of Helsinki. Written informed consent was obtained from all participants.

Studies I and II: Blood samples were taken in the morning after admission for investigating FA composition in serum in the patient group. Exclusion criteria for the patients were: a history of other psychiatric disorders than alcohol dependence, neurological illness and current or unstable medical illnesses or disabilities. Individuals on any kind of ongoing vitamin substitution were also excluded from the study. All patients were free of psychiatric medication at the time of FA determination. The healthy controls were free of medical or neuropsychiatric illnesses and were judged to be mentally healthy by an experienced psychiatrist. The average alcohol consumption (1 alcohol unit = 20 g alcohol/d), the duration and the severity of the alcohol abuse according to clinical impression were assessed. The liver enzymes Gamma-glutamyltransferase (GGT), Alanine-Aminotransferase (ALAT) and Aspartate-Aminotransferase (ASAT) were additionally analysed in the patients.

There were no differences related to age, gender, body mass index (BMI), the number of vegetarians, and the frequency of fish in the diet between both sub-groups. However, the alcoholic patients were significantly more often smokers and reported to drink substantially more units of alcohol per day than the controls (Table 1).

### Analysis of FAs

Total lipids were extracted from each 100 microliters blood-serum by means of chloroform/methanol 1:1 (v:v), dried under nitrogen. The FA extracts were then methylated using boron-trifluoride/methanol (14:86 vol:vol), and then re-extracted with neat pentane. All solvents were of HPLC grade purity. The concentration of a range of FA with chain-length of 14–22 C-atoms was measured by means of capillary gas-chromatography (HP 5890; pillar: Carbowax HP 20 M, detector: FID, oven temp. 180-250°C, rate 1°/min.). The following FAs were quantified (in brackets the trivial names and abbreviations used in the text) 14:0 (myristic acid; MYR); 16:0 (palmitic acid; PA); 16:1 (palmitoleic acid; PAI); 18:0 (stearic acid; STE); 18:1n-9 (oleic acid; OL); 18:2 n-6 (linoleic acid; LA); 18:3n-6 (γ-linolenic acid; GLA); 18:3n-3 (α-linolenic acid; ALA); 20:3n-6 (dihomo-γ-linolenic acid; DGLA); 20:3n-9 (mead acid; MEAD); 20:4n-6 (arachidonic acid; AA); 20:5n-3 (eicosapentaenoic acid; EPA); 22:6n-3 (docosahexaenoic acid; DHA). The relative concentration of each of these 13 serum FA was assigned as a percentage of the concentration [mg/l] of each FA divided by the sum of all 13 FA in mg/l. FA values were presented as percentage and absolute measures. FA standards of the Sigma Company were used for the quantitative and qualitative calibration, margaric acid (17:0) was used as internal standard. The coefficient of variation of the analytical method (CVM) was for each FA much lower than the coefficient of variation from patient to patient (CVP). [CVM of 14:0, 16:0, 16:1, 18:0, 18:1 and 18:2 ≤0.15; CVM of 18:3n-3, 18:3n-6, 20:3n-6, 20:4, 20:3n-9, 20:5 and 22:6 ≤ 0.22; CVP ≥ 0.6].

Blood of the patients and comparison group was collected between 9.00 and 10.00 a.m., after overnight fasting (the subjects had a quasi-empty bowel, only a fat-free light breakfast was permitted).

### Statistical methods

Variance analysis repeated measurement design was used to test for differences between the matched samples with all 13 FA concentrations or relative concentrations as dependent variables. Related samples Wilcoxon Rank test was applied to test for differences in the distribution of categorical variables between the samples or between before and after detoxification. One way ANOVA was calculated to analyse differences in FA concentrations between those patients with pathological liver enzyme concentrations and those with normal values. These associations were additionally tested by Spearman Rank correlation. The following ratios between various FA concentrations were also considered in the analysis: oleic acid concentration : linoleic concentration (Q1); Σ of all N-3 FA concentrations : Σ of all n-6 FA concentrations (Q2); and Σ of all MUFA concentrations : Σ of all poly-unsaturated FA (PUFA) concentrations (Q3). The significance level for all tests was set at p < 0.05. Because of the explorative nature of our study, we did not correct the level of significance for multiple testing. However, several results were highly significant (p < 0.001) and might remain significant even after a conservative correction for multiple testing, e.g. according to Bonferroni. Data were analysed using SPSS version 18 (SPSS Inc., Chicago, IL, USA).

## Results

### Study I – Comparison between alcohol dependent patients and healthy controls

The alcohol dependent patients reported an average history of alcohol abuse of about 9 years with a range from 1 to 31 years. About 32% of the patients presented themselves for detoxication without any breath alcohol (sober), while the other 68% exhibited an average breath alcohol concentration of 0.139 ± 0.089%, ranging from 0.011 to 0.306%.

### Differences in FA concentration between alcohol dependent patients and healthy controls

The absolute concentration of the FA was significantly higher in the patients compared to the healthy controls for PA, PAI, STE, OL, GLA, and ALA with the highest difference being for PAI followed by OL, whereas it was higher in the controls for DHA (see Table 2). Table 2 also gives the case numbers (n) and the power of the statistical tests (i.e. the probability that the null hypothesis will be rejected if the null hypothesis is false (type-II error)).

**Table 2:** **Differences between alcohol dependent patients and matched controls in FA concentration (mean ± sd in μg/ml) and their relative portion by variance analysis repeated measurement (Study I)**

	Alcoholic patients	Controls	Wilk’s λ	P	η.^2^	Power
MYR	23.25 ± 16.36	23.83 ± 23.20	1.00	0.892	<0.001	0.052
%	1.98 ± 0.79	3.07 ± 1.62	0.71	<0.001	0.292	0.986
PA	398.62 ± 241.02	264.29 ± 183.52	0.81	0.002	0.193	0.888
%	33.85 ± 2.82	32.17 ± 4.39	0.90	0.030	0.102	0.591
PAI	78.36 ± 52.67	30.56 ± 24.65	0.58	<0.001	0.419	1.00
%	6.52 ± 2.43	3.91 ± 1.21	0.49	<0.001	0.513	1.00
STE	89.81 ± 63.43	54.15 ± 35.23	0.76	0.001	0.236	0.950
%	7.64 ± 1.31	6.98 ± 1.22	0.86	0.010	0.143	0.755
OL	262.76 ± 153.53	144.81 ± 93.97	0.66	<0.001	0.340	0.996
%	22.32 ± 3.13	18.78 ± 1.97	0.53	<0.001	0.468	1.00
LA	232.48 ± 136.56	184.25 ± 114.09	0.92	0.061	0.077	0.468
%	20.25 ± 3.80	24.28 ± 4.16	0.59	<0.001	0.411	1.00
GLA	5.25 ± 3.83	3.61 ± 2.51	0.88	0.018	0.121	0.674
%	0.43 ± 0.19	0.49 ± 0.22	0.96	0.194	0.038	0.252
ALA	8.66 ± 6.84	5.03 ± 3.18	0.82	0.003	0.184	0.869
%	0.72 ± 0.24	0.70 ± 0.31	1.00	0.760	0.002	0.060
Mead	2.82 ± 2.65	1.99 ± 2.01	0.93	0.083	0.067	0.411
%	0.23 ± 0.10	0.29 ± 0.32	0.97	0.220	0.034	0.230
DGLA	12.54 ± 10.05	11.21 ± 7.63	0.99	0.496	0.011	0.103
%	1.04 ± 0.35	1.50 ± 0.52	0.64	<0.001	0.357	0.998
AA	45.68 ± 23.36	42.52 ± 28.69	0.99	0.496	0.011	0.105
%	4.12 ± 1.35	5.54 ± 1.58	0.66	<0.001	0.339	0.996
EPA	7.28 ± 6.02	6.57 ± 6.24	0.99	0.517	0.010	0.098
%	0.59 ± 0.32	0.89 ± 0.47	0.79	0.002	0.206	0.910
DHA	6.03 ± 4.52	10.82 ± 9.94	0.80	0.002	0.205	0.910
%	0.54 ± 0.25	1.40 ± 0.68	0.39	<0.001	0.606	1.00
Sum of FAs	1173.53 ± 671.12	783.63 ± 494.08	0.79	0.001	0.209	0.915
OL/LA	1.16 ± 0.34	0.80 ± 0.17	0.45	<0.001	0.546	1.00
ΣN-3 : ΣN-6	0.07 ± 0.02	0.10 ± 0.04	0.76	0.001	0.236	0.950
Σ MUFA : Σ PUFA	1.12 ± 0.36	0.68 ± 0.15	0.40	<0.001	0.598	1.00
OL % : LA %	1.16 ± 0.34	0.80 ± 0.17	0.48	<0.001	0.523	1.00
ΣN-3% : ΣN-6%	0.07 ± 0.02	0.10 ± 0.04	0.78	0.001	0.219	0.929
Σ MUFA% : Σ PUFA%	1.11 ± 0.36	0.68 ± 0.15	0.42	<0.001	0.580	1.00

The percentage of the various FAs in relation to the sum of all 13 analysed FAs was significantly higher in the patient group for PA, PAI, STE, and OL, whereas the relative concentration of MYR, LA, DGLA, AA, EPA, and DHA was higher in the healthy controls with the biggest difference for DHA followed by PAI. The total sum of FAs, the ratio OL/LA (Q1), and the ratio MUFA/PUFA (Q3) in blood serum were significantly higher in the patient than in the control group, both based on the absolute and on the relative values. In the opposite, the ratio n-3/n-6 FAs (Q2) was significantly lower in the patient than in the control group.

### Relationships between FA concentration and background variables

Age, BMI, smoking habits as well as the amount of fish in the diet were not significantly related to any absolute or relative FA concentration in both groups. However, the sum of all FAs in serum was significantly associated with age (R = 0.60; p <0.001); and the ratio Q1 (absolute: R = 0.45; p = 0.002; relative: R = 0.044; p = 0.003) as well as the ratio Q3 (absolute: R = 0.54; <0.001; relative: R = 0.055; p = <0.001) were in controls significantly correlated with the BMI for the absolute and relative concentrations. Age was significantly related to the ratio Q2 (R = 0.34; p = 0.024) in the patient group. There was no further significant association between the number of years of alcohol abuse and any FA concentration in the alcohol dependent patients.

### Associations between liver enzymes concentration and FAs’ concentration in alcohol dependent patients

Pathological ASAT concentrations were found in 47.1% of the patient group; 38.2% exhibited pathological ALAT values and 68.1% had a pathologic GGT score. The relative concentrations of PAI and OL were significantly higher in those alcohol dependent patients with a pathologically increased ASAT (F = 10.48; p = 0.003 and F = 8.60; p = 0.006, respectively) or GGT value (F = 4.43; p = 0.043 and F = 13.68; p = 0.001, respectively) than in those with a normal ASAT or GGT value. The absolute concentration of the FAs LA and AA (F = 7.10; p = 0.012 and F = 4.78; p = 0.036, respectively) was significantly higher in those patients with normal ASAT values compared to those with pathologically high values. Furthermore, the relative concentration for LA was higher in those patients with normal values in ASAT, ALAT, or GGT concentration (F = 13.65; p = 0.001, F = 5.44; p = 0.026 and F = 12.08; p = 0.001, respectively) compared to those with pathological values; and the relative concentration of AA was higher in patients with a normal ASAT value (F = 8.29; p = 0.007); that of LA was higher in patients with a normal ALAT value (F = 5.44; p = 0.026); and that of GLA was higher in patients with a normal GGT value (F = 5.76; p = 0.026) compared to those pathological concentrations of these FAs. The sum of all FAs in serum did not differ between those patients with pathologically high or normal liver enzyme concentrations. However, alcohol dependent patients with pathologically elevated liver enzyme concentrations showed significantly higher ratios Q1 and Q3 than the patients with normal liver enzyme concentrations for the absolute as well as for relative FA concentrations (Table 3).

**Table 3:** **Differences in FA ratios between groups with different liver enzyme concentration**

	ASAT		ALAT		GGT	
	F	P	F	P	F	P
OL: LA	13.09	0.001	5.13	0.030	15.73	< 0.001
OL % : LA%	12.99	0.001	5.06	0.031	15.59	< 0.001
MUFA : PUFA	15.95	< 0.001	5.06	0.032	15.28	< 0.001
MUFA % : PUFA %	15.80	< 0.001	5.08	0.031	15.06	< 0.001

Those patients with elevated liver enzyme concentration additionally had higher concentrations of alcohol in their breath at admission (ASAT: F = 19.13; p < 0.001, ALAT: F = 6.63; p = 0.015; and GGT: F = 5.32; p = 0.028). Furthermore, the concentration of alcohol was positively correlated with the relative concentration of PAI, and negatively with the relative concentration of the FAs LA, GLA, DGLA, and AA.

The only absolute FA concentration which was significantly correlated with liver enzyme concentrations was that for PA (ASAT: R = 0.42; p = 0.014; ALAT: R = 0.38; p = 0.025).

Substantial associations between all three determined liver enzyme concentrations and relative FA concentrations occurred for PAI, OL (positive), and LA (negative); whereas GGT concentration was additionally negatively related with the relative concentrations of GLA, DGLA, and AA. The concentrations of ASAT and ALAT were significantly correlated with the ratios of FAs Q1 %, Q2, Q2 %, Q3, and Q3 %. The ratios Q1, Q3, and Q3 % were additionally significantly associated with the GGT concentration, whereas the alcohol concentration was only substantially related to Q3 and Q3 % (Table 4).

**Table 4:** **Selected Spearman Rank correlations between relative FAs’ concentration and liver enzyme concentration (R (p))**

	ASAT	ALAT	GGT	Breath alcohol concentration
PAI %	0.61 (<0.001)	0.39 (0.021)	0.53 (0.001)	0.43 (0.012)
OL %	0.45 (0.008)	0.20 (0.089)	0.50 (0.003)	0.13 (0.477)
LA %	−0.59 (<0.001)	−0.43 (0.011)	−0.47 (0.005)	−0.36 (0.036)
GLA %	−0.25 (0.148)	−0.13 (0.470)	−0.43 (0.011)	−0.14 (0.425)
DGLA %	−0.31 (0.077)	−0.13 (0.448)	−0.34 (0.050)	−0.49 (0.004)
AA %	−0.34 (0.050)	−0.18 (0.305)	−0.50 (0.002)	−0.43 (0.012)
OL : LA	0.58 (<0.001)	0.39 (0.024)	0.52 (0.002)	0.32 (0.068)
OL : LA%	0.59 (<0.001)	0.39 (0.021)	0.52 (0.002)	0.31 (0.070)
Σ N-3 : Σ N-6	0.43 (0.011)	0.36 (0.038)	0.28 (0.115)	0.23 (0.182)
Σ N-3 : Σ N-6%	0.42 (0.014)	0.36 (0.036)	0.25 (0.151)	0.21 (0.234)
Σ MUFA : Σ PUFA	0.65 (<0.001)	0.42 (0.013)	0.61 (<0.001)	0.44 (0.009)
Σ MUFA : Σ PUFA %	0.64 (<0.001)	0.42 (0.013)	0.61 (<0.001)	0.44 (0.009)

### Study II – Comparison between alcohol dependent patients before and after detoxification

*Differences in FA concentration before and after detoxification from alcohol* The absolute concentrations of the FAs were significantly higher before compared to after detoxification for PA, PAI, STE, OL, GLA, ALA, AA, and EPA with highest differences for ALA followed by PA (see Table 5).

**Table 5:** **Differences before and after detoxification in FA concentration (mean ± sd in μg/ml) and their relative portion by variance analysis, repeated measurement (Study II)**

	Alcoholic patients before detoxification	Alcoholic patients after detoxification	Wilk’s λ	P	η.^2^	Power
MYR	27.73 ± 26.52	21.98 ± 14.42	0.95	0.068	0.051	0.447
%	2.25 ± 0.95	2.28 ± 1.07	1.00	0.811	0.001	0.056
PA	428.37 ± 322.80	303.15 ± 150.71	0.84	<0.001	0.162	0.958
%	34.40 ± 2.69	33.10 ± 3.30	0.86	0.001	0.143	0.928
PAI	104.60 ± 160.70	42.45 ± 31.89	0.84	<0.001	0.160	0.955
%	7.33 ± 2.90	4.56 ± 1.50	0.39	<0.001	0.614	1.00
STE	93.60 ± 58.05	78.91 ± 42.03	0.94	0.038	0.059	0.552
%	7.58 ± 1.30	8.46 ± 1.48	0.78	<0.001	0.217	9.993
OL	299.79 ± 283.12	187.13 ± 84.89	0.84	<0.001	0.156	0.950
%	23.60 ± 3.32	20.95 ± 2.34	0.63	<0.001	0.373	1.00
LA	211.92 ± 114.13	213.27 ± 103.41	1.00	0.922	<0.001	0.051
%	18.02 ± 4.12	23.52 ± 3.67	0.42	<0.001	0.577	1.00
GLA	4.84 ± 2.75	3.83 ± 2.54	0.92	0.013	0.084	0.715
%	0.42 ± 0.15	0.43 ± 0.30	1.00	0.751	0.001	0.061
ALA	8.53 ± 6.99	5.41 ± 2.58	0.83	<0.001	0.166	0.962
%	0.69 ± 0.23	0.70 ± 0.75	1.00	0.850	0.001	0.054
Mead	2.96 ± 2.32	2.54 ± 1.75	0.97	0.160	0.027	0.289
%	0.24 ± 0.11	0.27 ± 0.14	0.96	0.095	0.038	0.387
DGLA	10.53 ± 5.32	11.34 ± 6.97	0.99	0.327	0.081	0.164
%	0.92 ± 0.29	1.22 ± 0.39	0.68	<0.001	0.325	1.00
AA	44.49 ± 25.36	36.62 ± 22.40	0.92	0.014	0.081	0.702
%	3.77 ± 1.31	4.01 ± 1.32	0.98	0.183	0.024	0.263
EPA	7.29 ± 6.24	4.94 ± 3.51	0.87	0.001	0.133	0.906
%	0.59 ± 0.30	0.59 ± 0.51	1.00	0.965	<0.001	0.050
DHA	5.55 ± 3.73	4.90 ± 3.95	0.98	0.185	0.024	0.262
%	0.47 ± 0.23	.51 ± 0.22	0.98	0.250	0.18	0.208
Sum of FAs	1255.36 ± 982.64	916.18 ± 435.15	0.87	0.002	0.126	0.889
OL : LA	1.44 ± 0.63	0.92 ± 0.22	0.58	<0.001	0.419	1.00
ΣN-3 : ΣN-6	0.08 ± 0.02	0.06 ± 0.02	.65	<0.001	0.346	1.00
Σ MUFA : Σ PUFA	1.40 ± 0.68	0.87 ± 0.22	0.58	<0.001	0.421	1.00
OL % : LA %	1.44 ± 0.63	0.92 ± 0.22	0.58	<0.001	0.419	1.00
ΣN-3 % : ΣN-6 %	.08 ± 0.02	0.06 ± 0.03	0.87	0.002	0.129	0.895
Σ MUFA% : Σ PUFA%	1.40 ± 0.68	0.86 ± 0.22	0.57	<0.001	0.427	1.00

The percentage of PA, PAI, STE and OL in relation to the sum of all 13 analysed FAs was significantly higher before compared to after detoxification, whereas the relative concentration of LA and DGLA was lower before detoxification. There was no significant difference in the relative concentrations of GLA, ALA, DGLA, AA, and EPA the absolute concentration of LA and in both absolulate and relative concentrations of MEAD and DHA before and after detoxification. All three ratios Q1, Q2, Q3 based on the absolute and the relative concentrations as well as the total sum of FAs were significantly higher before compared to after detoxification.

However, when controlling the one-way ANOVAs for age, alcohol concentration, years of alcohol abuse, number of days of in-patient treatment, and number of average alcohol units per day (as co-variates), none of the tested comparisons remained significant. Age was the variable with the most substantial impact on these correlations indicated by the “re-occurrence” of significant differences in most FA concentrations when age was excluded from the list of co-variate variables.

### Relationships between FA concentration and background variables

The amount of fish in the diet, the years of alcohol abuse, the average number of alcohol units drunken per day, BMI, and age were not significantly related to any absolute or relative FA concentration at both assessment time points. Smokers had lower absolute MYR, GLA, DGLA, and EPA concentrations before detoxification (F = 18.05; p < 0.001; F = 5.41; p = 0.023; F = 11.20; p = 0.001; F = 6.57; p = 0.012, respectively) and lower absolute PA, PAI, LA, and DHA concentrations after detoxification (F = 4.58; p = 0.036; F = 8.19; p = 0.006; F = 4.37; p = 0.040; F = 10.35; p = 0.002, respectively). Furthermore, Q2 based on the relative concentrations was lower in smokers before detoxification (F = 7.49; p = 0.008). The alcohol concentration at admission was significantly correlated with the concentrations of MYR, PA, PAI, OL, GLA, and ALA (F = 0.30; p = 0.017; F = 0.31; p = 0.008; F = 0.43; p < 0.001; F = 0.36; p = 0.002; F = 0.026; p = 0.028; F = 0.33; p = 0.004, respectively) as was the relative concentration of ALA (F = 0.27; p = 0.21).

### Associations between liver enzyme and FA concentrations

Pathologically increased ASAT concentrations were found in 58.9% of the alcohol dependent patients; 53.4% exhibited increased ALAT concentrations and 78.1% had an increased GGT score. The relative concentration of PA was higher in those alcohol dependent patients with an ASAT concentration within the normal range before (F = 18.79; p < 0.001) and after detoxification (F = 5.71; p = 0.019) compared to those with pathological ASAT concentration; whereas the relative concentration of PAI was higher in patients with a pathological, i.e. increased ASAT concentration (before: F = 20.90; p < 0.001; after: F = 7.39; p = 0.006). The absolute concentration of PAI (F = 4.17; p = 0.045) and ALA (F = 4.23; p = 0.043) as well as the relative concentration of OL (F = 6.69; p = 0.012) and ALA (F = 4.39; p = 0.040) were higher before detoxification in those with an increased ASAT concentration; but the relative concentration of MYR was higher in those with a normal ASAT value (F = 7.22; p = 0.009) only before detoxification.

The relative PAI concentration was higher in patients with increased ALAT or GGT concentrations at both assessments (before: F = 8.13; p = 0.006; F = 4.83; p = 0.031, respectively; after: F = 9.47; p = 0.003; F = 6.03; p = 0.016, respectively); whereas the relative PA concentration was higher in patients with normal ALAT or GGT scores only before detoxification (F = 10.43; p = 0.002; F 4.78; p = 0.032, respectively).

The ratio between the two FA concentrations Q2 and Q3 was significantly higher in patients with an increased ASAT concentration before detoxification (absolute: F = 10.60; p = 0.002; relative: F = 10.13; p = 0.002; absolute: F = 5.48; p = 0.022; relative: F = 5.45; p = 0.022, respectively), but not after detoxification. The ratio between the FA concentrations Q1 and Q3 was significantly higher in patients with an increased ALAT concentration only after detoxification (absolute: F = 4.67; p = 0.020; relative: F = 4.63; p = 0.035; absolute: F = 5.16; p = 0.026; relative: F = 5.07; p = 0.027, respectively). Those with an increased GGT concentration did not differ from patients with a normal GGT value in any FA based ratio.

Furthermore, the concentration of ASAT and GGT was significantly correlated with age (R = 0.24; p = 0.042; R = 0.23; p = 0.048, respectively), and the ASAT concentration was correlated with the alcohol concentration (R = 0.30; p = 0.009).

### Study I and Study II

The concentrations of all FAs (absolute and relative values) as well as all three analysed FA ratios that significantly differed between alcohol dependent patients and controls (Study I) were found to be closer to the control levels in the patients after detoxification (Study II) indicating a tendency towards normalisation of the FA concentrations during the course of detoxification (lower absolute and relative values for PA, PAI, STE, OL, Q1, Q2, and Q3; only for absolute values for GLA, ALA, AA, EPA, and the sum of all FAs; higher relative concentration for LA and DGLA). However, when comparing the FA concentrations of the controls from Study I with the concentrations of the patients after detoxification (t-tests for independent samples), the patients still showed higher absolute and relative concentrations for PAI (absolute: t = 2.14; p = 0.035; relative: t = 2.44; p = 0.016), STE (absolute: t = 3.30; p = 0.001; relative: t = 5.62; p < 0.001), and OL (absolute: t = 2.53; p = 0.013; relative: t = 5.17; p < 0.001), and lower values for DHA (absolute: t = 3.82; p < 0.001; relative: t = 10.48; p < 0.001). Furthermore, the relative concentrations were still higher in the controls for MYR, DGLA, AA, and EPA (t = 2.88; p = 0.005; t = 3.32; p = 0.001; t = 5.68; p < 0.001; t = 3.15; p = 0.002, respectively). The ratios Q1 and Q3 were still significantly higher in the patients than in the controls (absolute: t = 3.46; p = 0.001; t = 5.59; p < 0.001, respectively; relative: t = 3.44; p = 0.001; t = 5.43; p < 0.001, respectively), whereas the ratio Q2 was still higher in the controls (absolute: t = 6.35; p < 0.001; relative: t = 4.88; p < 0.001).

## Discussion

The PUFA composition of the cell membrane lipids is most important for its fluidity and permeability. The embedded FAs affect the membrane function dependent on their degree of (de)saturation. A gammalinolenicacid-enriched diet reduces the microviscosity and increases the unsaturation index of microsomal membranes in rats [23]. An unweighted PUFA composition might therefore contribute to a dysfunctional metabolism observed in the central nervous system of alcohol dependent patients [24]. Changes of the FA metabolism due to nutritional behaviour are reflected in serum concentrations much faster than in membranes or adipose tissue [25]. Therefore (and because of accessibility and convenience), we used serum and not membranes as substrate to analyse FA concentrations during the course of short-term alcohol detoxification, assuming that the changes in serum FAs may have a connection to their membrane composition.

Alcoholism often is related to poorer nutritional uptake of FAs. Decreased nutrient densities of saturated, monounsaturated, polyunsaturated, linoleic, and alpha-linolenic acids have been associated with increasing alcohol consumption in alcohol dependent men [26]. In our study, the habits of fish consumption and vegetarism did not substantially differ between patients and controls; but, the alcohol dependent individuals did significantly more often use tobacco.

### MUFAs

The most robust characteristic of serum FA composition of untreated alcoholic patients is the increase of oleic acid at the cost of linoleic acid. Normally, in the whole serum the predominant FA of the C18 group is linoleic acid (unpublished data). An elevated level mono-unsaturated FAs (MUFA) in plasma was observed in rats after chronic ethanol feeding [27]. Furthermore, the percentage of MUFA was higher in white and brown adipose tissues of alcohol-treated rats, compared to control animals without alcohol exposure [28]. The presence of ethyl oleate (besides other FA ethylic esters) increased by more than 10 fold in mouse liver extracts after alcohol administration [29]. In individuals with so-called intermittent explosive disorder, who all had alcohol abuse problems, the concentration of Linoleic acid, the precursor of the n-6 FAs, was below normal, while oleic acid was elevated in plasma [30].

### PUFAs

Maturu et al. [31] examined the relation of FA composition of erythrocyte membrane phospholipids with the plasma lipid profile and other plasma metabolites in patients suffering from chronic alcoholism in comparison with healthy controls. They found changes in the erythrocyte membrane of patients, namely higher palmitic acid (saturated) and lower special n-3 FAs. Alcohol-induced FA alterations in plasma and erythrocyte membranes were assumed to be an adaptive response in order to counteract the deleterious effects of alcohol.

DHA is particularly vulnerable to oxidative damage in ethanol withdrawal [32].

No significant differences were found between a group of 80 alcohol dependent patients with oral supplementation of 2 g PUFAS for 3 months and a placebo group regarding the reduction of the amount of alcohol ingestion; further measured parameters in this placebo/controlled, double blind, randomized study were patients’ craving and alcohol dependence severity scores [24]. The capsules contained n-3 and n-6 PUFAs in a ratio of approximately 3.5 : 1.

Our results confirm those of Rosnowska et al. [9] who examined 25 alcohol-dependent patients before and after clinical detoxification; the authors found an increased oleic acid level accompanied by a decrease in linoleic acid and in total FAs before detoxication, probably due to the stimulation with hydrogen excess formed during alcohol fermentation. Therefore, it might be speculated that the ratio of oleic (MUFA)/ to linoleic acid (n-6 PUFA) discriminates better than n-3/n-6 PUFAs between the metabolism during acute alcohol intoxication and normalized metabolism.

### Mechanism

The mechanism by which chronic ethanol consumption reduces the concentrations of PUFAs was examined by Pawlosky et al. [33]: In livers of alcohol dependent men, more radioactive labelled linolenic and 22:5 n-3 acid were utilized for the synthesis of EPA and DHA, than was predicted from plasma kinetics. This ability to utilize linolenic acid for the synthesis of longer chained PUFA was greater in alcohol dependent individuals than in controls.

From animal experiments it is known, that heavy alcohol consumption increases the synthesis of FAs in the liver and mobilizes the peripheral triglycerides from the adipose tissue into the liver [34]. In rats, the unsaturation of fatty acyl chains after alcohol feeding increased in the liver, whereas it decreased in plasma [35].

Oleic acid constitutes the main FA component in the human serum lipid fraction of triglycerides (unpublished data). After the breakdown of triglyceride molecules in the liver, oleic acid may be the source for the de novo-synthesis of FAs. Similarly, oleic acid is the main FA component in the serum free FAs (unpublished data).

Warensjö et al. [36] suggested that serum FA composition reflects the endogenous FA synthesis catalysed by Delta-desaturases despite dietary fat intake. They described an effect, similar to the alcohol-induced shifts in oleic/ linoleic acid in our study, in the cholesteryl esters of men with metabolic syndrome and suggested that serum “FA composition predicts the long-term development of the metabolic syndrome, and Delta-5 Desaturase activity may be particularly important in this process. The possibility that altered FA composition, partly secondary to genetic or hormonal factors, should also be considered” [36].

Another reason for the high oleic concentration may be the Stearoyl-Coenzyme A desaturase-1. The delta-9 FA desaturase Stearoyl-Coenzyme A desaturase-1 converts saturated FAs into monounsaturated fatty acids (MUFA) and this activity is elevated by dietary carbohydrate [37].

Changes in the distribution of saturated and unsaturated FAs in the plasma phospholipid FA composition could indicate a disturbance of FA metabolism [27].

Heavy ethanol use blocks FA oxidation through inhibitions of peroxisome proliferator-activated receptor-α and of AMP-activated protein kinase. Under certain conditions, the de novo synthesis of FA in the liver can be increased by ethanol feeding [34]. Additionally, acid sphingomyelinase activity in plasma was found to be (reversibly) increased in patients with alcohol-dependence and to correlate with lipoproteins [38]. In sphingomyelins, oleic acid is bound in high concentrations.

## Conclusion

Serum FA composition is influenced by alcohol abuse. It is mainly characterised by a shift from linoleic to oleic acid as the quantitatively predominant C 18 acid. After detoxification, a normalization towards lower OL and higher LA acid takes place. Possibly, the higher OL production serves as a shelter mechanism against the damage of free radicals by alcohol ingestion, since OL prevents among others TNF-α induced oxidative stress mediated cardiomyocyte cell damage [39]. Dietary intake of polyunsaturated fats increases the probability of liver injuries in response to ethanol feeding; however, increased levels of oleic acid and lower levels of linoleic acid may lead to attenuated liver injuries after toxine exposure [40]. OL and moderate wine consumption are components of a so-called Mediterranean diet which by some is believed to be protective against cancer and cardiovascular diseases [41]. We assume that OL contributes to alleviate the adverse reactions of alcohol consumption. OL accumulation in serum seems to be a special effect of alcohol: in the liver of mice with a non-alcoholic fatty liver disease, a low concentration of OL was found [42].

In summary, our study provides insight in FA pattern changes during long-term and heavy alcohol misuse compared to healthy controls. Contrary to our expectation, the most striking changes were not only decreased concentrations of essential PUFAs in the patient group before detoxification, but also increased MUFA concentrations (OL, PAI), thus these represent possible trait markers. The influence of pre-existing liver damages and different “lifestyle” factors must also be taken into consideration. Further studies are needed in order to corroborate our findings; liver enzymes should also be measured in controls and additionally female individuals should be examined as well in order to uncover possible gender differences.

## Abbreviations

AA: Arachidonic acid (20:4n-6)
ALA: α-linolenic acid (18:3n-3)
DGLA: Dihomo-γ-linolenic acid (20:3n-6)
DHA: Docosahexaenoic acid (22:6n-3)
EPA: Eicosapentaenoic acid (20:5n-3)
FA: Fatty acids
GLA: γ-linolenic acid (18:3n-6)
LA: Linoleic acid (18:2 n-6)
MEAD: Mead acid (20:3n-9)
MUFA: Mono unsaturated fatty acids
MYR: Myristic acid (14:0)
OL: Oleic acid (18:1n-9)
PA: Palmitic acid (16:0)
PAI: Palmitoleic acid (16:1)
PUFA: Poly unsaturated fatty acids
Ratio Q1: oleic acid concentration : linoleic concentration
Ratio Q2: Σ of all N-3 fatty acids concentration : Σ of all N-6 fatty acids concentrations
Ratio Q3: Σ of all MUFA concentrations : Σ of all PUFA concentrations
STE: stearic acid (18:0).
